# Supplementary material for: Cognitive Sequelae and Hippocampal Dysfunction in Chronic Kidney Disease following 5/6 Nephrectomy
Source: Brain Sci. 2022 Jul 11;12(7):905. doi: 10.3390/brainsci12070905 (PMC9321175; doi:10.3390/brainsci12070905)
Supplement: Supplementary file 1 [file brainsci-12-00905-s001.zip › brainsci-1784870-supplementary.pdf]

Supplementary Methods

Serum biochemical assays

All serum biochemical assays were performed using the following commercially available kits. Blood urea nitrogen (BUN) was measured by a specific quantitative colorimetric assay (Quantichrome Urea Assay Kit) from BioAssay Systems according to the manufacturer's protocol. Serum creatinine was measured by another quantitative colorimetric assay (Creatinine Reagent Set) from Pointe Scientific following the manufacturer's instructions.

Novel objective recognition test

The induction and maintenance of activity depend upon the physical presence of an object, which is used to detect the difference in responses (recognition) to a novel physical object in a familiar place. The novel object recognition test used a black acrylic box (60 × 60 × 40 cm in the open field test and allowed the animal to become habituated to the box a day before the test for 30 minutes. In the habituation phase the next day, rats from all groups were placed back in the same chamber, which contained two similar objects. The rats were allowed to freely explore the objects for a total of 10 minutes and then, were returned to their home cages. In the test phase 24 h later, one of the objects was replaced with a novel object with a different shape and color. The rats were placed back in the test chamber with the novel object and one previously explored object (familiar object) for 10 minutes. In each phase, the animals were placed at the center area of the box facing the wall that was opposite two objects. Reading and analysis of the video were manually performed. The object-exploring behaviors were analyzed including accessing, sniffing, biting, and direct contact with the forelimb. The rats were evaluated for their ability to remember the familiar object. Object exploration was defined as the time the rats were in direct contact with the object (e.g., sniffing the object, < 1 cm from the object). The discrimination index (DI) of the NOR test was the time spent exploring the novel object compared to the familiar object and expressed as the ratio of novel object exploration time to total exploration time for both objects: DI = (novel object exploration time - familiar object exploration time)/total exploration time.

Y-maze with special cue

Three arms of the Y-maze (40 x 5 x 20 cm; length, width, height) were indicated A, B, C and positioned at equal angles, converged at an angle of 120°. Each animal was placed at the end of one arm and allowed to navigate freely for an 8-min session. During the 8-min period, the sequence (e.g., ABC, BCA, CAB) and the number of arm entries were recorded manually for each animal. Spontaneous alternation behavior was defined as entry into all three arms as consecutive choices (e.g., ABC, CAB, or BCA but not ABA). The maze arms were thoroughly cleaned between tasks to remove residual odor. The percentage (%) of spontaneous alternation behavior was defined according to the following equation: % alternation = [(number of alternations)/(total arm entries − 2)] × 100.

Barns maze

Four different visual cues were placed on each quadrant wall surrounding the platform at a height that the animals could observe easily. One hole (the escape hole) of the quadrant zone contained an escape chamber under the platform. Bedding was added to the escape chamber in which rats could hide for safety. A metronome (80 Hz noise) and high-lighting (300 lux of illumination at the center of the platform) were used to amplify anxiety for motivation to find an escape chamber. The animals interacted with the Barnes maze in three phases: habituation (1 day), training (2 – 4 days in long training paradigms, and probe (1 day). During the adaptation phase, which lasted four days, the rats were placed in a black cylinder at the center of the platform. After 10 sec, when the cylinder was removed, the animals explored the platform from the center and the activity was recorded by a video camera. The rats were given 180 sec to find the escape hole. When a rat entered the escape chamber within 180 sec, a cover was placed on the hole for 120 sec to block the light and stop the electronic sound. Otherwise, the animals were carefully pulled and guided to the hole with the escape chamber to induce learning about the space. To enhance the spatial memory for finding the escape hole, each animal was tested 3 times at 15 min intervals during the acquisition-training phase. On the fifth day, which was the spatial acquisition phase (probe phase), the animals were given 90 seconds to find the escape hole, but the escape chamber had been removed. This phase was performed only once, versus three times in the adaptation phase. The time (latency) to reach the escape chamber

was recorded and analyzed.

Classical fear conditioning test

Each test consisted of a training phase, followed 24 h later by the testing phase. During the training phase, the rats were individually placed into a conditioning chamber inside a sound-proof box and allowed to explore for 3 min, after which, a conditioned stimulus (CS) (an 80-dB tone) was played for 20 sec. The last 2 sec of the CS was paired with a 0.5-mA foot-shock. The mice were given three tone-shock pairings at 1-min intervals and then returned to their home cage. Twenty-four hours after training, contextual fear memory was evaluated by placing the animal into the same training context and measuring freezing behavior for 5 min. Freezing, defined as a complete lack of movement besides respiration, was used as an indication of learning in this task. The data are expressed as the percentage of freezing during each minute or as a mean across all minutes.

fEPSP *in vivo*

Animals (naïve, 4 and 10 wks after surgery) were intraperitoneally anesthetized using urethane 1.5 g/kg and placed into a stereotaxic frame. Rectal temperature was maintained at 37 ± 0.3 °C during the course of the surgery using a temperature controller (Harvard Instruments, USA). The scalp was opened and separated. Holes were drilled through the skull for introducing electrodes. The coordinates (in mm) referenced to the bregma were as follows. For the recording electrode in adult rats (to the Schaffer collateral): 4.0 mm posterior to the bregma, 3.0 mm lateral to the midline, at 2.5 mm depths. For the stimulating electrode (to the stratum radiatum of CA1): 3.5 posterior to the bregma, 2.0 lateral to the midline, at 3.5 depths. Electrode depths were finally determined by optimizing the evoked response. fEPSPs were adjusted to ～60% of the maximal response for testing. Stimulation was generated by a BNC-2110 apparatus (National Instruments, USA) and a Digital Stimulus Isolation unit (Getting Instruments, USA). Pyramidal neuron responses to Schaffer collateral stimulation were recorded with a P55A.C. pre-amplifier (3–1000 Hz bandpass, Astro-Med Inc.) and analyzed using WinLTP ver 2.01 software (WinLTP Ltd.). Responses were evoked by single-pulse stimuli and delivered at 20-sec intervals. A stable baseline was recorded for 30 – 60 min. Long-term potential (LTP) was induced by strong theta-patterned stimuli (sTPS, four trains of 10 bursts of 5

pulses at 400 Hz with a 200-ms inter-burst interval and 15-sec inter-train interval) because sTPS (bursts of 400 Hz stimuli) are NMDA-receptor dependent. Robust LTP of the CA1 and dentate gyrus (DG) areas were evoked by sTPS *in vivo*. To analyze the changes in fEPSP, fEPSP slopes were averaged over 60-sec intervals and expressed as percentages of the mean fEPSP slope measured during the 30-min baseline period, which was expressed as 100%. The fEPSP responses of the naïve animal were measured using the same method.

Hippocampal volume in patients with cognitive impairment

To measure the volume of the hippocampus, 3D T1-weighted volumetric images were taken using the following parameters: FOV = 256 x 256 mm, voxel size = 0.98 × 0.98 × 1.2 mm^3, TR=8.7ms, TE=4.6, and flip angle=8°. Automated volume measurement of the hippocampus was made using Inbrain® (IB, https://www.inbrain.co.kr/index.html) software. IB is a Korean FDA-cleared software based on the FreeSurfer platform enhanced with its own deep learning algorithm. The IB segmentation method is similar to the FreeSurfer method, which is based on volumetric- and surface-based segmentation and uses a template-driven approach. The IB processing included analysis failure prediction, intensity normalization, brain extraction, registration into volume and surface atlas, white matter segmentation, white matter surface smoothing, topology correction, pial and white matter surface optimization, comparisons between the output results and the database, and an analysis of quality management. Finally, the volume of regional brain structures and cortical thickness was obtained. A deep learning algorithm was applied to multiple steps, including analysis failure prediction, brain extraction, white matter segmentation, and the analysis of quality management to enhance the quality of the segmentation results. The entire process took about four hours.

Quantification of data and statistical analysis

Optical fractionation was used to estimate cell numbers. Optical fractionation (combination counting with optical dissection with fractionator sampling) is a stereological method based on a properly designed systematic random sampling method that, by definition, yields unbiased estimates of population numbers. The sampling procedure is

accomplished by focusing through the depth of the tissue (the optical dissector height, *h;* of 30 μm in all cases for this study). The number of each cell type (C) in each subregion was estimated by: C = $\Sigma Q-$ × t/h × 1/asf × 1/ssf, where $Q-$ is the number of cells actually counted in the dissectors that fell within the sectional profiles of the subregion seen on the sampled sections, and asf is the areal sampling fraction calculated by the area of the counting frame of the dissector, a(frame) (50 × 50 μm^2 in this study) and the area associated with each x, y movement grid (x, y step) (of 250 × 250 μm^2 in this study) {asf = [a(frame)/a(x,y step)]}. ssf is the fraction of the sections sampled or section sampling fraction [minimum of six tissues per each animal model in this study, since we used all sections in the entire hippocampus]. The immunoreactive cells were counted with a 40× objective lens. All immunoreactive cells were counted regardless of labeling intensity. Cell counts were performed by two different investigators who were blinded to the tissue classifications. For immunodensity quantification, the CA1 and DG regions were delineated with a 2.5× objective lens. Each image was normalized by adjusting the black and white range of the image using Adobe PhotoShop v. 8.0. Thereafter, 10 areas per rat (250 μm^2 for each area) were selected and intensity measurements are presented as the mean 256 grayscale number (using NIH Image 1.59 software). Values for background staining were obtained from the corpus callosum. Optical density values were corrected by subtracting the average background noise values obtained from five image inputs.
